# GLP-1 Receptor Agonist NLY01 Reduces Retinal Inflammation and Neuron Death Secondary to Ocular Hypertension

**DOI:** 10.1016/j.celrep.2020.108271

**Published:** 2020-11-03

**Authors:** Jacob K. Sterling, Modupe O. Adetunji, Samyuktha Guttha, Albert R. Bargoud, Katherine E. Uyhazi, Ahmara G. Ross, Joshua L. Dunaief, Qi N. Cui

**Affiliations:** 1 FM Kirby Center for Molecular Ophthalmology, Scheie Eye Institute, University of Pennsylvania Perelman School of Medicine, Philadelphia, PA 19104, USA; 2 Medical Scientist Training Program, Perelman School of Medicine, University of Pennsylvania, Philadelphia, PA 19104, USA; 3 Present address: Grossman School of Medicine, New York University, New York, NY 10016, USA; 4 Present address: Rutgers New Jersey Medical School, Newark, NJ 07103, USA; 5 Lead Contact; 6 Twitter: @jacobksterling; 7 Twitter: @cuilab

## Abstract

Glaucoma is the leading cause of irreversible blindness and is characterized by the death of retinal ganglion cells (RGCs). Recent studies have implicated pro-inflammatory microglia, macrophages, and A1 astrocytes in the pathogenesis of neurodegenerative diseases. The role of pro-inflammatory, neurotoxic A1 astrocytes in glaucoma is just beginning to be explored. Using a mouse model of glaucoma, we demonstrate that ocular hypertension is sufficient to trigger production of C1q, interleukin-1α (IL-1α), and tumor necrosis factor α (TNF-α), three cytokines necessary and sufficient to drive the formation of A1 astrocytes. Upregulation of these cytokines occurs first in CD11b^+^ CD11c^+^ cells followed by CD11b^+^ CD11c^−^ cells. Ablation of this pathway, by either genetic deletions of C1qa, IL-1α, and TNF-α, or treatment with glucagon-like peptide-1 receptor agonist NLY01, reduces A1 astrocyte transformation and RGC death. Together, these results highlight a neuroinflammatory mechanism of glaucomatous neurodegeneration that can be therapeutically targeted by NLY01 administration.

## In Brief

Sterling et al. show that in response to ocular hypertension, CD11b^+^ CD11c^+^ cells contribute to early retinal inflammation and astrogliosis in a mouse model of glaucoma. Ablation of this inflammatory pathway, via genetic deletion or administration of the GLP-1R agonist NLY01, prevents astrogliosis and retinal ganglion cell death.

## Graphical Abstract

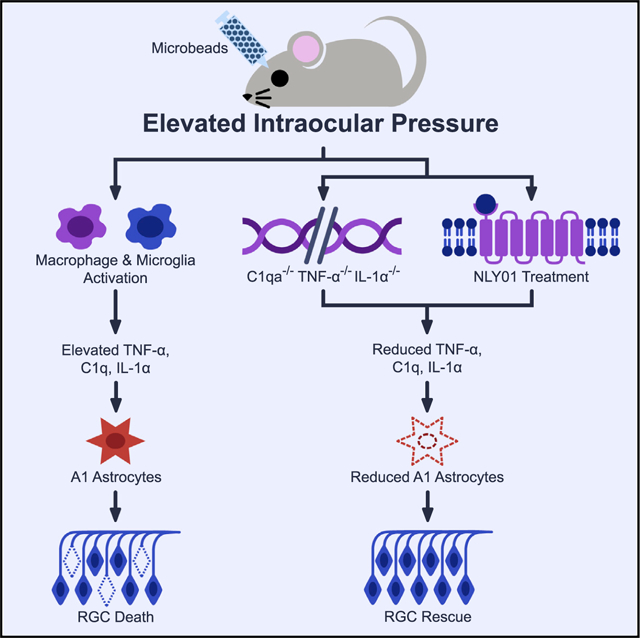

## INTRODUCTION

Glaucoma is characterized by the death of retinal ganglion cells (RGCs), leading to permanent vision loss. It is the leading cause of irreversible blindness globally and is projected to affect ~112 million people worldwide by 2040 (Tham et al., 2014). Elevated intraocular pressure (IOP) is strongly associated with glaucoma, and reduction of IOP is the only therapeutic mechanism available to slow disease progression. However, glaucoma can continue to progress even in patients who achieve normal IOPs following medical and/or surgical treatments (Quigley, 2019). Therefore, new therapies are urgently needed to prevent vision loss in patients with glaucoma.

Reactive astrocytes are observed in multiple neurodegenerative diseases (Liddelow et al., 2017; Yun et al., 2018). In healthy neural tissue, astrocytes serve a wide variety of roles. They contribute to neurotransmitter recycling, neuronal metabolism, and formation of the blood-brain and blood-retina barriers (Clarke and Barres, 2013; Liddelow and Barres, 2017). In the retina, astrocytes are found exclusively in the ganglion cell layer, comingled with RGCs (Vecino et al., 2016). In response to both local and systemic stimuli, astrocytes can adopt reactive forms, A1 pro-inflammatory or A2 neuroprotective, both of which have been transcriptionally defined (Zamanian et al., 2012). A1 reactive astrocytes lose their phagocytic capacity as well as their ability to promote synapse formation and function. At the same time, A1 astrocytes gain pro-inflammatory and neurotoxic functions (Liddelow et al., 2017; Zamanian et al., 2012). In contrast, A2 astrocytes, observed in post-ischemic tissue, upregulate neurotrophic factors, promoting a neuroprotective environment (Zamanian et al., 2012). While A1 astrocytes have been implicated in multiple neurodegenerative diseases (Liddelow et al., 2017; Yun et al., 2018), their contribution to glaucoma is only beginning to be explored.

In the brain and retina, neurotoxic A1 astrocytes are induced by microglial release of pro-inflammatory cytokines interleukin-1α (IL-1α), tumor necrosis factor α (TNF-α), and C1q (Liddelow et al., 2017). Strong links exist between these three cytokines and glaucoma. *IL1A* and *TNF* polymorphisms are associated with primary open angle glaucoma (Bozkurt et al., 2012; Fan et al., 2010; Mookherjee et al., 2010; Wang et al., 2006). TNF-α protein levels are elevated in the vitreous, retina, and optic nerves of glaucomatous eyes (Williams et al., 2017). In the DBA/2J mouse model of hypertensive glaucoma, *C1qa* mRNA levels are associated with disease progression (Stevens et al., 2007), and C1q inhibition is sufficient to prevent early RGC synapse loss and RGC death (Howell et al., 2011, 2014; Williams et al., 2016). Multiple publications have also demonstrated C1q upregulation in glaucomatous human eyes (Reinehr et al., 2016; Stasi et al., 2006). Although IL-1α, TNF-α, and C1q have been independently implicated in glaucoma, it is not known whether they act in concert to induce A1 astrocyte reactivity in glaucomatous retinas.

Glucagon-like peptide 1 is an incretin hormone that regulates blood glucose, weight, and satiety through its action at the glucagon-like peptide 1 receptor (GLP-1R) in both the systemic circulation and the central nervous system (Drucker, 2018). NLY01 is a long-acting GLP-1R agonist with an extended half-life and favorable blood-brain barrier penetration (Yun et al., 2018). In mouse models of Parkinson’s disease (PD), A1 astrocytes contribute to dopaminergic cell death and poor motor phenotypes. NLY01 has been shown to reduce microglial production of C1q, TNF-α, and IL-1α, thereby blocking A1 astrocyte transformation, reducing dopaminergic cell death, and improving motor symptoms in mouse models of PD (Yun et al., 2018).

Using the microbead-induced ocular hypertension mouse model of glaucoma, we show that microglia and infiltrating macrophages upregulate C1q, TNF-α, and IL-1α. These three cytokines are necessary for A1 transformation within the retina. Cytokine upregulation and A1 transformation persist despite normalization of IOP 6 weeks post-injection. Genetic deletion of these cytokines prevents A1 astrocyte formation and RGC loss. Finally, NLY01 therapy reduced CD11b^+^ CD11c^−^ and CD11b^+^ CD11c^+^ production of C1q, TNF-α, and IL-1α; A1 astrocyte transformation; and RGC loss in our model. Together, these data demonstrate that GLP-1R activation is capable of reducing ocular inflammation driven by both CD11b^+^ CD11c^−^ and CD11b^+^ CD11c^+^ cell populations, thus preventing A1 astrocyte activation and rescuing RGCs from hypertensive glaucoma. NLY01 has potential clinical use in the treatment of glaucoma and possibly other retinal diseases characterized by reactive astrogliosis.

## RESULTS

### Elevated IOP Induces A1 Astrocyte Reactivity in the Retina

**Figure 1.**
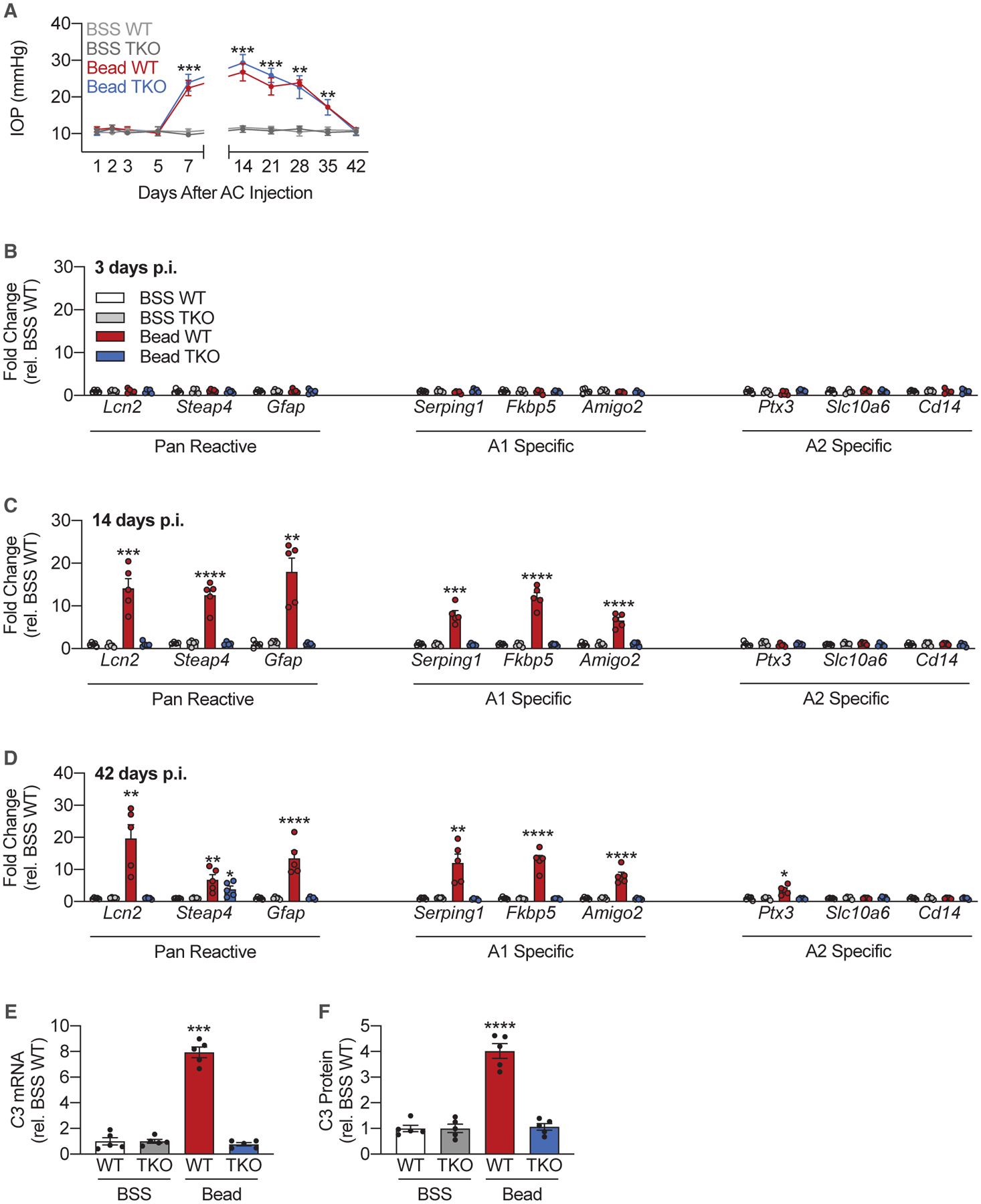
Elevated IOP Induces A1 Astrocyte Reactivity in the Retina C57BL6/J (WT) and *Il1a*^−/−^; *Tnf*^−/−^; *C1qa*^−/−^ knockout (TKO) mice were injected either with microbeads (“Bead,” left eye) into the anterior chamber (AC) to increase intraocular pressure (IOP) or with BSS (right eye). (A) IOP measurements across the duration of the study in both eyes of WT and TKO animals. The statistical difference for Bead WT versus BSS WT and Bead TKO versus BSS TKO is shown using asterisks. No statistical difference was detected between BSS WT and BSS TKO or Bead WT and Bead TKO (n = 20 eyes per condition per genotype). (B–D) qPCR measurements of pan-reactive, A1-specific, and A2 specific transcripts from ACSA2^+^ cells isolated from WT and TKO mice at 3 days (B), 14 days (C), and 42 days (D) post-injection (p.i.) (n = 5 eyes per condition per genotype). (E) qPCR measurements of *C3* mRNA levels in ACSA2^+^ cells 42 days post-injection (n = 5 eyes per condition per genotype). (F) ELISA measurements of C3 protein levels in ACSA2^+^ cells 42 days post-injection (n = 5 eyes per condition per genotype). All data are presented as mean ± SEM. *p < 0.05, **p < 0.01, ***p < 0.001, and ****p < 0.0001 versus BSS WT (Mann-Whitney *U* test). See also Figure S1.

Magnetic microbeads (left eye) or balanced salt solution (BSS; right eye) were injected into the anterior chamber (AC) of wild-type (WT) and *Il1a*^−/−^; *Tnf*^−/−^; *C1qa*^−/−^ triple knockout (TKO) mice. IOP was recorded at 1, 2, 3, 5, and 7 days post-injection and weekly thereafter. Microbead-injected eyes in both WT and TKO animals had elevated IOPs (eIOPs) beginning at 7 days post-injection compared to BSS-injected eyes (Figure 1A). IOPs peaked at 14 days post-injection and remained elevated throughout the 35 days post-injection. There was no difference in IOP between microbead- and BSS-injected eyes by the 42nd day post-injection (Figure 1A). Neurosensory retinas were isolated for cell sorting 3, 14, and 42 days post-injection (Figure S1A). Cell-type enrichment for all fractions used in the paper were validated by qPCR (Figures S1B and S1C). Astrocytes and Müller cells were isolated using ASCA^2+^ selection, as reported previously (Kantzer et al., 2017). By the 14th day post-injection, ACSA2^+^ cells exhibited increased levels of pan-reactive and A1-astrocyte-specific markers (Figures 1B–1D), suggesting that A1 astrocyte transformation occurs early in the disease process. A1 reactivity persisted in microbead-injected WT animals 42 days post-injection, despite normalization of IOP (Figure 1D). TKO animals failed to form A1 astrocytes despite eIOP (Figures 1C and 1D). This is consistent with previous findings (Guttenplan et al., 2020; Liddelow et al., 2017). Markers of A2 reactivity were consistently unchanged in WT microbead-injected eyes compared to WT BSS-injected eyes (Figures 1C and 1D).

Liddelow et al. (2017) demonstrated that complement component 3 (C3) is a marker for A1 astrocytes (Liddelow et al., 2017). We measured C3 mRNA and protein levels in ACSA2^+^ cells isolated from both BSS- and microbead-injected WT and TKO retinas 42 days post-injection. C3 mRNA (Figure 1E) and protein (Figure 1F) levels were elevated in WT microbead-injected eyes compared to both BSS-injected eyes and TKO microbead-injected eyes (Figures 1E and 1F). These data suggest that C3 production in ACSA2^+^ cells, which encompass both astrocytes and Müller cells, is dependent on IL-1α, TNF-α, and C1q and that loss of A1 reactivity reduces C3 production.

### TNF-α, IL-1α, and C1q Contribute to RGC Death Secondary to eIOP

**Figure 2.**
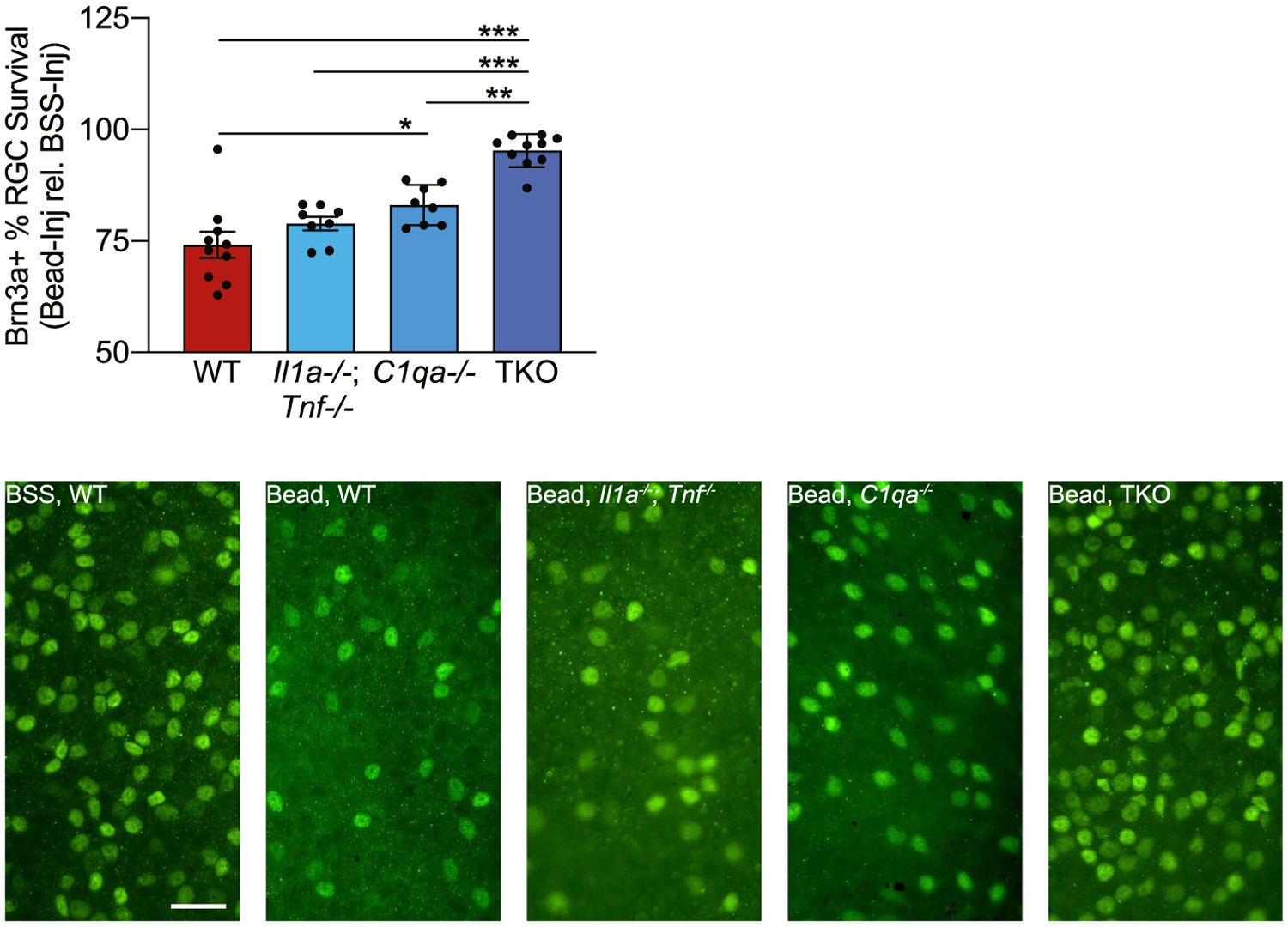
TNF-α, IL-1α, and C1q Trigger RGC Death Secondary to eIOP C57BL6/J (WT), *Il1a*^−/−^; *Tnf*^−/−^, *C1qa*^−/−^, and *Il1a*^−/−^; *Tnf*^−/−^; *C1qa*^−/−^ knockout (TKO) mice were injected either with microbeads (left eye) into the AC to increase IOP or with BSS (right eye). At 42 days post-injection, mice were euthanized and retinal flatmounts were stained for the retinal ganglion cell (RGC) marker Brn3a. The cell count for each microbead-injected eye was normalized against the contralateral BSS-injected eye to determine percent survival of Brn3a^+^ cells. n = 10 mice per genotype. All data are presented as mean ± SEM. *p < 0.05, **p < 0.01, and ***p < 0.001, one-way ANOVA with Tukey’s multiple comparisons test. Representative images are shown on the right. Scale bar, 50 mm.

To determine whether TNF-α, IL-1α, and C1q transformation of A1 astrocytes plays a role in RGC death in the microbead-induced eIOP model of glaucoma, WT, *Il1a*^−/−^; *Tnf*^−/−^ double knockout (DKO) mice, *C1qa*^−/−^ single knockout mice, and *Il1a*^−/−^; *Tnf*^−/−^; *C1qa*^−/−^ (TKO) mice were injected with magnetic microbeads in one eye and BSS in the fellow eye. After 42 days, whole retina flatmounts were stained for the RGC marker Brn3a (Nadal-Nicolás et al., 2009). Each data point (Figure 2) represents the RGC count in the microbead-injected eye divided by the RGC count in the BSS-injected eye of the same mouse, multiplied by 100. There was no difference in RGC loss observed in DKO mice compared to WT mice. There was a modest improvement in RGC survival observed in *C1qa*^−/−^ mice compared to WT mice, consistent with previously published results (Howell et al., 2011, 2014; Williams et al., 2016). RGC death was reduced in TKO mice compared to all other genotypes (Figure 2), suggesting that the loss of all three cytokines in combination provided an additional benefit beyond the loss of either IL-1α and TNF-α or C1q alone.

### Early Retinal Inflammation Is Driven by CD11b^+^ CD11c^+^ Cells and Persists beyond IOP Re-normalization

**Figure 3.**
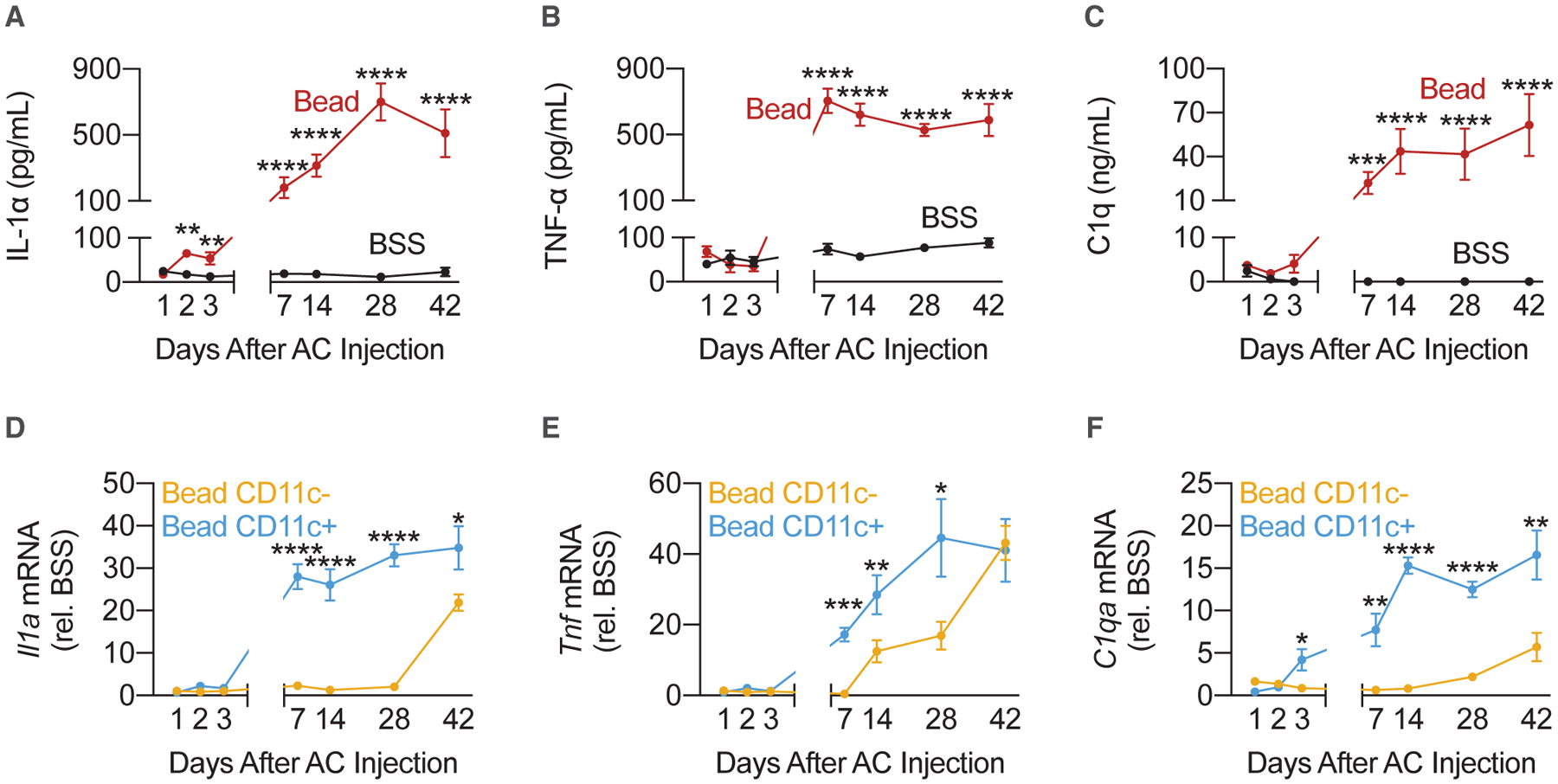
Early Retinal Inflammation Is Driven by CD11b^+^ CD11c^+^ Cells and Persists Beyond Re-normalization of IOP C57BL6/J (WT) mice were injected either with microbeads (“Bead,” left eye) into the AC to increase IOP or with BSS (right eye). (A–C) ELISA measurements of IL-1α (A), TNF-α (B), or C1q (C) protein levels in whole neurosensory retina at 1, 2, 3, 5, 7, 14, 28, and 42 days post-injection. Statistical test compared BSS-injected eyes to microbead-injected eyes at the same time points (n = 5 eyes per time point per condition). (D–F) CD11b^+^ CD11c^−^ and CD11b^+^ CD11c^+^ cells were isolated from neurosensory retina at 1, 2, 3, 5, 7, 14, 28, and 42 days post-injection. *Il1a* (D), *Tnf* (E), and *C1qa* (F) mRNA levels were measured by qPCR in both cell populations. qPCR measurements in microbead-injected eyes were normalized to the contralateral BSS-injected eyes. Statistical tests compared microbead-injected CD11b^+^ CD11c^+^ cells and microbead-injected CD11b^+^ CD11c^−^ cells at the same time points (n = 5 eyes per time point per condition). All data are presented as mean ± SEM. *p < 0.05, **p < 0.01, ***p < 0.001, and ****p < 0.0001, Mann-Whitney *U* test. See also Figures S1–S3.

To address the time course and source of IL-1α, TNF-α, and C1q production, we injected either microbeads or BSS into the AC of WT mice. Neurosensory retinas were isolated 1, 2, 3, 7, 14, 28, and 42 days post-injection and were either used to measure IL-1α, TNF-α, and C1q protein levels by ELISA or dissociated for cell sorting. IL-1α, TNF-α, and C1q protein levels increased in line with IOP, rising by day 7 and then plateauing at subsequent time points (Figure 3A–3C). IL-1α, TNF-α, and C1q remained elevated 6 weeks post-injection, despite a return of IOP to baseline levels (Figures 1A and 3A–3C). Microbead-injected eyes that did not have a significant increase in IOP, observed in ~5% of eyes, did not have elevated IL-1α, TNF-α, or C1q mRNA levels in CD11b^+^ cells 42 days post-injection (Figures S2A–S2C). Together, these data suggest that the retinal inflammatory response, initiated by ocular hypertension, can outlast IOP elevation.

Previous studies have implicated CD11b^+^ cells in the production of IL-1α, TNF-α, and C1q (Liddelow et al., 2017; Yun et al., 2018). Recent work in the DBA/2J mouse model of glaucoma has further refined our understanding of CD11b^+^ cell subpopulations. In the DBA/2J mouse model, early inflammation is driven by CD11b^+^ CD11c^+^ cells while CD11b^+^ CD11c^−^ cells adopt a largely anti-inflammatory pattern of gene expression (Tribble et al., 2019). We isolated CD11b^+^ CD11c^+^ and CD11b^+^ CD11c^−^ cells (Figure S3A) and measured *Tmem119* mRNA levels in both populations (Figure S3B), a marker of resident microglia (Bennett et al., 2016). We compared the expression of *Il1a*, *Tnf*, and *C1qa* between CD11b^+^ CD11c^−^ and CD11b^+^ CD11c^+^ cells isolated from microbead-injected eyes. Both sets of microbead-injected eyes were normalized against BSS-injected eyes. CD11b^+^ CD11c^+^ cells exhibited earlier expression of *Il1a*, *Tnf*, and *C1qa* than CD11b^+^ CD11c^−^ cells (Figures 3D–3F). Expression of *Il1a*, *Tnf*, and *C1qa* by CD11b^+^ CD11c^−^ cells also increased but lagged behind those of CD11b^+^ CD11c^+^ cells by days to weeks (Figures 3D–3F). These results suggest that CD11b^+^ CD11c^+^ provided the primary source of pro-inflammatory signals in early glaucomatous retinal inflammation following eIOP. In further support of this hypothesis, we demonstrated that A1 astrocytes were present by 14 days post-injection (Figures 1B–1D), when CD11b^+^ CD11c^−^ cells did not express all three cytokines necessary for A1 transformation (Figures 3D–3F). Therefore, initial A1 transformation is unlikely to be driven by CD11b^+^ CD11c^−^ cells in this model.

### NLY01, a GLP-1R Agonist, Reduces IL-1α, TNF-α, and C1q Production by CD11b^+^ CD11c^+^ and CD11b^+^ CD11c^−^ Cells and Decreases A1 Astrocyte Activation during eIOP

NLY01, a GLP-1R agonist, has been shown to modulate microglial phenotype reducing A1 astrocyte activation in the brain in a GLP-1R-dependent manner (Yun et al., 2018). We hypothesized that NLY01 therapy would reduce IL-1α, TNF-α, and C1q production by both microglia and macrophages, thereby decreasing A1 astrocyte conversion secondary to eIOP. To test the efficacy of NLY01 in our model, we used microbead injections to induce eIOP (Figure S4). Mice were given twice-weekly subcutaneous injections of either NLY01 at a dose of 5 mg/kg or normal saline solution (NSS). Neurosensory retinas were harvested 14 and 42 days post-injection to evaluate the effects of NLY01 on both the CD11b^+^-CD11c^+^-mediated early response and the CD11b^+^-CD11c^−^-mediated late response. NLY01 had no effect on IOP in microbead- or BSS-injected eyes (Figure S4).

**Figure 4.**
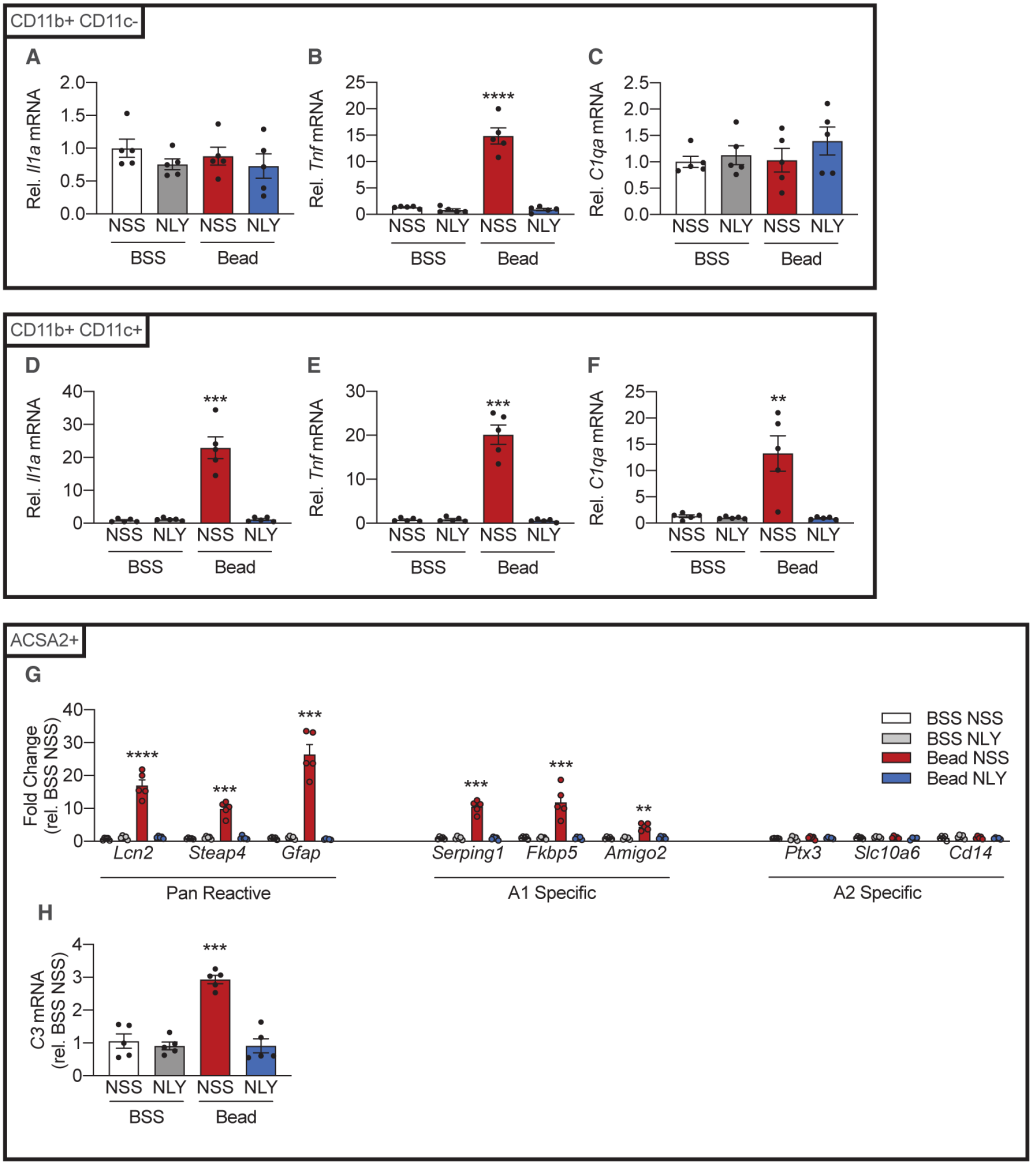
The GLP-1R Agonist NLY01 Reduces Production of IL-1α, TNF-α, and C1q Secondary to eIOP and A1 Astrocyte Activation on the 14th Day Post-injection C57BL6/J (WT) mice were injected either with microbeads (“Bead,” left eye) to increase IOP or with BSS (right eye). Following intraocular injections, mice were randomized to twice-weekly subcutaneous NLY01 (5 mg/kg per injection) or normal saline. Mice were euthanized 14 days post-injection. (A–F) CD11b^+^ CD11c^−^ and CD11b^+^ CD11c^+^ cells were isolated from neurosensory retina. qPCR was performed to measure *Il1a* (A and D), *Tnf* (B and E), and *C1qa* (C and F) mRNA levels in each population (n = 5 eyes per condition). (G) qPCR measurements of pan-reactive, A1-specific, and A2 specific transcripts from ACSA2^+^ cells 14 days post-injection (n = 5 eyes per condition). (H) qPCR measurement of *C3* mRNA levels in ACSA2^+^ cells. (n = 5 eyes per condition) All data are presented as mean ± SEM. *p < 0.05, **p < 0.01, ***p < 0.001, and ****p < 0.0001 relative to BSS NSS (Mann-Whitney *U* test). See also Figures S1, S4, and S5.

By the 14th day post-injection, NLY01 therapy reduced CD11b^+^ CD11c^−^ (resident microglia enriched) upregulation of TNF-α (Figure 4B) without altering basal expressions of IL-1α or C1q (Figures 4A and 4C). NLY01 also reduced CD11b^+^ CD11c^+^ expression of IL-1α, TNF-α, and C1q (Figures 4D–4F). In the ACSA2^+^ (astrocyte- and Müller-cell-enriched) fraction, NLY01 also reduced expression of pan-reactive transcripts, A1-specific transcripts, and C3, consistent with decreased A1 activation (Figures 4G and 4H).

**Figure 5.**
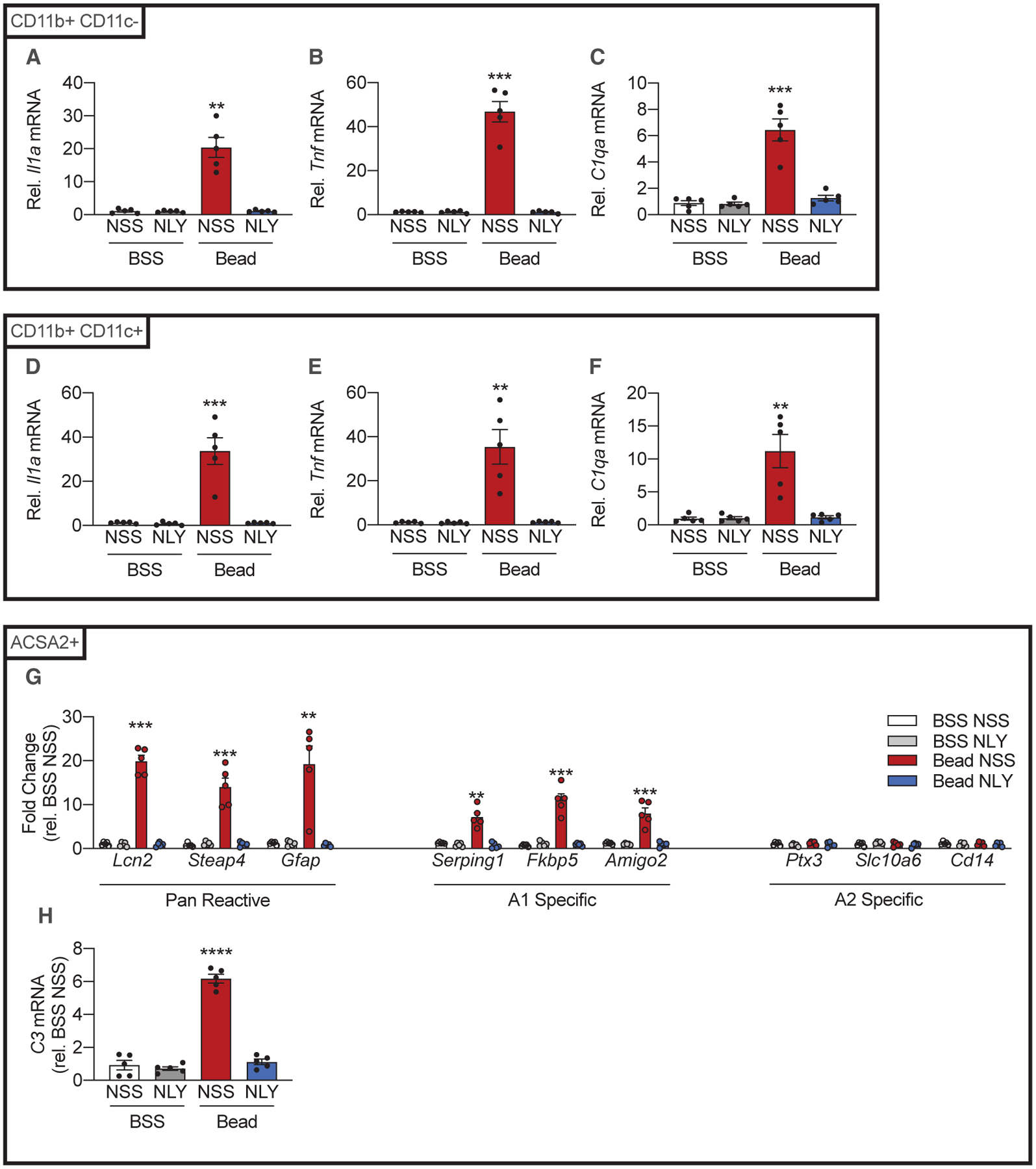
NLY01 Reduces CD11b^+^ CD11c^+^ and CD11b^+^ CD11c^−^ IL-1α, TNF-α, and C1q Production and A1 Astrocyte Activation on the 42nd Day Post-injection C57BL6/J (WT) mice were injected either with microbeads (“Bead,” left eye) to increase IOP or with BSS (right eye). Following injections, mice were randomized to twice-weekly subcutaneous NLY01 (5 mg/kg per injection) or normal saline. Mice were euthanized 42 days post-injection. (A–F) CD11b^+^ CD11c^−^ and CD11b^+^ CD11c^+^ cells were isolated from neurosensory retina. qPCR was performed to measure *Il1a* (A and D), *Tnf* (B and E), and *C1qa* (C and F) mRNA levels in each population (n = 5 eyes per condition). (G) qPCR measurements of pan-reactive, A1-specific, and A2 specific transcripts from ACSA2^+^ cells 42 days post-injection (n = 5 eyes per condition). (H) qPCR measurement of *C3* mRNA levels in ACSA2^+^ cells (n = 5 eyes per condition). All data presented as mean ± SEM. Mann-Whitney *U* test, **p < 0.01, ***p < 0.001, ****p < 0.0001 versus BSS NSS. See also Figures S1, S4, and S5.

By the 42nd day post-injection, NLY01 therapy reduced both CD11b^+^ CD11c^−^ (resident microglia enriched) and CD11b^+^ CD11c^−^ expression of IL-1α, TNF-α, and C1q (Figures 5A–5F). NLY01 also reduced expression of pan-reactive transcripts, A1-specific transcripts, and C3 in the ACSA2^+^ (astrocyte- and Müller-cell-enriched) fraction (Figures 5G and 5H), as it had by the 14th day post-injection (Figures 4G and 4H).

NLY01 has been shown to reduce the nuclear translocation and phosphorylation of the pro-inflammatory transcription factor nuclear factor κB (NF-κB) in a GLP-1R-dependent manner in brain microglia (Yun et al., 2018). At the 42nd day post-injection, CD11b^+^ CD11c^−^ and CD11b^+^ CD11c^+^ cells isolated from microbead-injected, NSS-treated eyes exhibited an increase in NF-kB protein phosphorylation (Figures S5A and S5B). NLY01 treatment decreased phosphorylated NF-κB protein levels (Figures S5A and S5B) and increased mRNA levels of IκBα (Figures S5C and S5D), a negative regulator of NF-κB and a known target of GLP-1R (Athauda and Foltynie, 2016). Together these data suggest that NLY01 modulates microglial/macrophage inflammatory phenotype via GLP-1R.

### NLY01 Reduces RGC Death Secondary to eIOP

**Figure 6.**
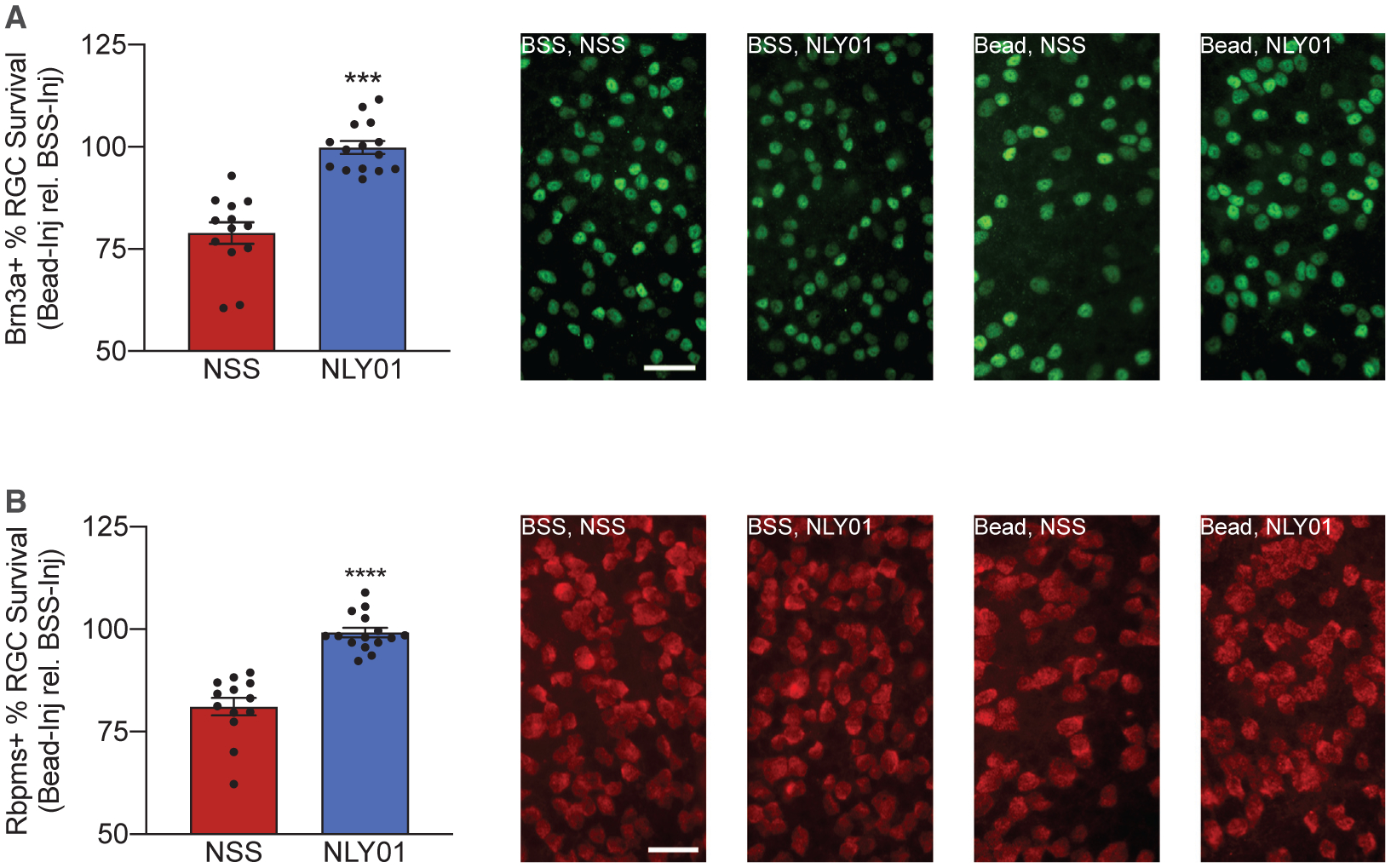
NLY01 Reduces RGC Death Secondary to eIOP C57BL6/J (WT) mice were injected either with microbeads (“Bead,” left eye) to increase IOP or with BSS (right eye). Following injection, mice were randomized to twice-weekly subcutaneous NLY01 (5 mg/kg per injection) or normal saline. Mice were euthanized 42 days post-injection. (A) Retinal flatmounts were stained for Brn3a. The cell count for each microbead-injected eye was normalized against the contralateral BSS-injected eye to determine percent survival of Brn3a^+^ cells. Representative images are shown on the right. Scale bar, 50 μm. (B) Retinal flatmounts were stained for Rbpms. Cell count for each microbead-injected eye was normalized against the contralateral BSS-injected eye to determine percent survival of Rbpms^+^ cells. Representative images are shown on the right. Scale bar, 50 μm. All data are presented as mean ± SEM; n = 13 NSS and n = 15 NLY01. ***p < 0.001 and ****p < 0.0001 versus BSS WT (Mann-Whitney *U* test). See also Figure S4.

To test the efficacy of NLY01 as a potential neuroprotective agent in our model, eIOP was once again induced through microbead injections of mice treated with either twice-weekly NLY01 at a dose of 5 mg/kg or NSS. After 42 days, neurosensory retinas were isolated, flat-mounted, and labeled with RGC markers Brn3a (Figure 6A) and Rbpms (Figure 6B) for RGC counting. Each data point (Figures 6A and 6B) represents the RGC count in the microbead-injected eye divided by the RGC count in the BSS-injected eye of the same mouse, multiplied by 100. NLY01 therapy reduced RGC death secondary to eIOP, as quantified by RBPMS^+^ and Brn3a^+^ immunofluorescence and cell counting (Figures 6A and 6B).

## DISCUSSION

Glaucoma is a neurodegenerative disease with potentially severe visual implications. Therapies to slow disease progression are currently limited to IOP reduction through both medical and surgical means. Unfortunately, successful reduction of IOP does not prevent disease progression in a significant number of patients. New therapies targeting other risk factors for glaucoma are needed to prevent irreversible vision loss. We examined the role of A1 reactive astrocytes in the microbead-induced ocular hypertension mouse model of glaucoma. Following induction of ocular hypertension, IL-1α, TNF-α, and C1q production was initially driven by CD11b^+^ CD11c^+^ cells. The contribution of CD11b^+^ CD11c^−^ cells to IL-1α, TNF-α, and C1q production was not observed until weeks to months after ocular injection. Together, these three cytokines triggered the formation of A1 astrocytes as demonstrated by upregulation of A1-specific transcripts and C3 production in an ACSA2^+^ retinal cell population (enriched for astrocytes and Müller cells). Treatment with the GLP-1R agonist NLY01 reduced microglia/macrophage production of IL-1α, TNF-α, and C1q; decreased A1 astrocyte conversion; and protected against RGC death in this mouse model of glaucoma.

Recent work by Guttenplan et al. (2020) corroborates several critical findings in this study. Using both optic nerve crush and microbead injections, they demonstrate that *Il1a*^−/−^; *Tnf*^−/−^; *C1qa*^−/−^ (TKO) mice exhibit significant reductions in RGC death at rates comparable to our findings. Further, preserved RGCs are functionally intact under examination by *in vivo* electrophysiology (Guttenplan et al., 2020). In combination, these results highlight the neurotoxic role of IL-1α, TNF-α, and C1q in RGC death following injury. Guttenplan and colleagues’ finding that rescued RGCs remain functionally viable lends further credence to inhibition of A1 astrocyte transformation as a possible therapy for glaucoma.

Transcriptomic data from the DBA/2J mouse model of glaucoma suggest that early inflammation is driven by CD11b^+^ CD11c^+^ cells, while CD11b^+^ CD11c^−^ cells initially adopt an anti-inflammatory pattern of gene expression (Tribble et al., 2020a; Williams et al., 2019). Our results support this finding by demonstrating that CD11b^+^ CD11c^+^ cells upregulated IL-1α, TNF-α, and C1q expression prior to contribution from CD11b^+^ CD11c^−^ cells. Results suggest that CD11b^+^ CD11c^+^ cells are early contributors to A1 astrocyte formation following IOP elevation (eIOP). Transcriptomic data from fluorescence-activated cell sorting (FACS)-isolated CD11b^+^ CD11c^+^ retinal cells in the DBA/2J mouse model of glaucoma demonstrate that this population is enriched for infiltrating macrophages compared to other blood-borne immune cells and resident microglia (Tribble et al., 2020a). It should be noted that the embryonic origin of the CD11b^+^ CD11c^+^ cell population has not been conclusively demonstrated, and these cells could therefore represent resident retinal microglia that have undergone a state change, infiltrating macrophages, or a mixture of both. However, macrophage infiltration has been implicated in the pathogenesis of glaucoma. Specifically, macrophages have been observed in sections of human glaucomatous retina and optic nerve in both mild and severe cases (Margeta et al., 2018). Progression of visual field loss in normotensive glaucoma is also associated with increased systemic levels of macrophage chemoattractant protein-1 (MCP-1), a potent chemotactic factor for monocytes (Lee et al., 2017). Although the presence of the blood-retina barrier confers a degree of immune privilege to the retina, disruption of the blood-retina barrier has been observed in diseases of ocular inflammation (Daruich et al., 2018; Kaur et al., 2008; Kokona et al., 2018; Vecino et al., 2016). Glaucomatous retinas often exhibit focal bleeds in the nerve fiber layer surrounding the optic nerve head. These so-called Drance hemorrhages disrupt the blood-retina barrier and present an opportunity for blood-borne immune cells, such as macrophages, to enter the retina (Williams et al., 2017). Together, these data provide an impetus for future work characterizing the origin of CD11b^+^ CD11c^+^ cells in mouse models of glaucoma as well as the role of infiltrating macrophages and the integrity of the blood-retina barrier in glaucoma.

Following microbead injections, the time course of IL-1α, TNF-α, and C1q upregulation corresponds to the trajectory of IOP increase, lending support to eIOP as the initiator of inflammation. Despite a return to normal IOP, pro-inflammatory cytokines remained upregulated at 6 weeks post-injection, suggesting that the inflammatory pathway remains active beyond the inciting eIOP. In human glaucomatous eyes, a reduction in IOP, whether by pharmacological or surgical means, is not always sufficient to prevent further RGC degeneration. Our data suggest that persistent inflammation after normalization of IOP may contribute to these refractory cases, presenting a treatment opportunity for patients who have exhausted therapies rooted in IOP reduction.

A1 astrocytes upregulate C3 (Liddelow et al., 2017), and C3 inhibitors were shown to reduce RGC cell death in the DBA/2J mouse model of glaucoma (Bosco et al., 2018). We demonstrate elevated C3 production following A1 activation and decreased C3 production following NLY01 inhibition in our glaucoma model. Importantly, C3 is not the only source of toxicity from A1 astrocytes (Liddelow et al., 2017). While C3 inhibition offers some RGC protection, prevention of A1 astrocyte formation may confer more complete protection against eIOP.

Within 2 weeks of microbead injection, pro-inflammatory CD11b^+^ CD11c^+^ cells upregulate IL-1α, TNF-α, and C1q expression, which triggers A1 astrocyte transformation. NLY01 blocks this pathway and rescues RGCs from eIOP-induced death at 6 weeks post-injection. Previous work demonstrates that RGC loss does not occur in the microbead model until after 4–6 weeks of prolonged eIOP (Calkins et al., 2018; Ito et al., 2016; Sappington et al., 2010). This delay between A1 transformation and RGC death raises the question of whether RGC-autonomous mechanisms of cell stress must act in concert with the non-cell-autonomous mechanism of retinal inflammation to trigger RGC death. Loss of one of these two pathways, conferred by NLY01 administration in our study, was sufficient to rescue RGCs. Several observations support this multi-hit hypothesis. First, in animal models of unilateral glaucoma, where one eye has eIOP and the other eye with normal IOP is used as an internal control, microglial activation and inflammation can be observed sans RGC loss in the control optic nerve (Tribble et al., 2020b). Second, neuronal injury, via either optic nerve crush or eIOP, is a necessary precursor for astrocyte-mediated neuroinflammatory cell death (Guttenplan et al., 2020). Targeting both RGC-autonomous mechanisms of stress and retinal inflammation may act in a synergistic fashion to rescue additional RGCs.

Elevated IOP induces deficits in axon transport along the optic nerve (Lambert et al., 2017, 2020) and reduction in NaV1.2 protein levels in RGCs (Risner et al., 2020) after 4 weeks of ocular hypertension. In contrast, changes in RGC electrical signaling occur earlier after eIOP. Following just 2 weeks of eIOP, RGCs exhibit increased electrical responses to preferred stimuli in both light onset and offset cells. During this same 2-week period, RGCs exhibit excessive dendritic pruning (Risner et al., 2018). In both the brain and the retina, dendritic pruning is linked to the production of C1q and the subsequent initiation of the classical complement cascade by CD11b^+^ cells (Stevens et al., 2007). Our results show that CD11b^+^ CD11c^+^ cells, and not CD11b^+^ CD11c^−^ cells, are responsible for C1q upregulation and possibly downstream dendritic pruning. NLY01 reduced *C1qa* expression in CD11b^+^ CD11c^+^ cells 2 weeks after IOP elevation, suggesting that NLY01 may also prevent synaptic pruning in RGCs. A1 astrocytes themselves may also directly promote aberrant electrical signaling, as *in vitro* work has shown that A1 astrocytes reduced the number of synapses, miniature excitatory postsynaptic current (mEPSC) frequency, and mEPSC amplitude in cultured RGCs (Liddelow et al., 2017).

In our study, microbeads were injected into the AC of one mouse eye while the contralateral eye was injected with BSS. This paired approach reduced the impact of biological variability through the use of an internal control. However, recent work has shown that unilateral induction of eIOP in rats resulted in microglial reactivity throughout the visual pathway, including in the contralateral, normotensive optic nerve (Tribble et al., 2020b). While most of our experiments normalized the microbead eye against the normotensive control eye, one exception to this can be found in Figures 3A–3C, where absolute protein levels were measured using ELISA. Here, normotensive eyes showed no upregulation of IL-1α, TNF-α, and C1q proteins, suggesting that if present, microglial reactivity in the control eye may not result in A1 astrocyte activation in this model of glaucoma.

NLY01 reduces microglial/macrophage activation and prevents A1 astrocyte formation. NLY01 belongs to a family of GLP-1R agonists. NLY01 is a long-acting GLP-1R agonist that efficiently penetrates the blood-brain barrier (Yun et al., 2018). In mouse models of PD, NLY01 concentrations in the brain are higher than in WT animals. The authors attributed this increase in NLY01 concentration to blood-brain barrier breakdown present in the PD mouse models (Yun et al., 2018). Similarly, blood-retina barrier disruptions in glaucoma (Williams et al., 2017) may serve a therapeutic benefit by providing a gateway for systemically administered therapeutics to access the retina and the optic nerve. This further highlights the need to characterize the state of the blood-retina barrier in glaucoma.

Previous work has shown that NLY01’s effects on CD11b^+^ cells are not limited to reductions in levels of IL-1α, TNF-α, and C1q. Rather, NLY01 treatment reduces microglia density and relative IBA1 levels in a mouse model of PD, suggesting a broader immunosuppressive effect (Yun et al., 2018). It is therefore possible that the RGC rescue we observed following NLY01 treatment can be partially attributed to a reduction in microglial/macrophage reactivity and occurs independent of the drug’s effect on astrocyte phenotype. This is consistent with findings from other groups demonstrating that reduced CD11b^+^ cell reactivity protects RGCs from the effects of eIOP (Williams et al., 2017).

The safety of NLY01 in humans is currently being tested in a clinical trial for PD. NLY01 belongs to the GLP-1R class of therapeutic agonists that have been used in the clinic for over 15 years. During that time, GLP-1R agonists have demonstrated a favorable safety profile in the long-term treatment of type 2 diabetes mellitus (Aroda, 2018). Diabetes is a known risk factor in glaucoma (Khan et al., 2017), and GLP-1R agonists’ wide usage in diabetes treatment presents an opportunity to retrospectively evaluate its effects among patients with coexisting glaucoma in a large-scale observational study. The findings of such a study could provide evidence as to whether existing GLP-1R agonists affect glaucoma incidence or progression among patients with diabetes.

Glaucoma is a group of diseases with disparate but often inter-linking etiologies. Our data highlight neuroinflammation as a mechanism of glaucomatous damage and demonstrate rescue by NLY01 through the drug’s ability to decrease retinal inflammation. Current therapies targeting IOP are not sufficient to prevent vision loss in many glaucoma patients. NLY01, or more broadly the class of GLP-1R agonists, may be a fruitful avenue for future exploration.

### Limitations of Study

RGC degeneration occurs in retrograde fashion, beginning with the retraction of RGC synaptic terminals in the colliculus, followed by axonal degeneration, with loss of RGC soma constituting a late step in the degenerative cascade (Buckingham et al., 2008). Our study does not include optic nerve analysis, and it is important that readers consider alternative explanations for these findings given this limitation. Rbpms and, to a lesser degree, Brn3a immunolabeling captures most, but not all, RGC somas, and it is possible that rescue is more robust than indicated by soma analysis alone. Conversely, it is possible that RGC counts underestimated RGC death by counting RGC soma that have yet to degenerate. It is also possible that ablation of A1 astrocytes prolonged expression of RGC markers in the soma leading to overcounting of RGCs in TKO and NLY01-treated animals. It should be noted that the rescued RGCs in TKO animals have been shown to be electrically and functionally intact (Guttenplan et al., 2020). NLY01 treatment decreases IL-1α, TNF-α, and C1q expression in a similar fashion to TKO, lending credence to the possibility that it rescues RGCs and preserves their functionality in a similar fashion. Nevertheless, we cannot say definitively that this is the case following NLY01 treatment until we have evaluated the optic nerve, which we plan to pursue in future experiments.

## STAR★METHODS

### RESOURCE AVAILABILITY

#### Lead Contact

Further information and requests for resources and reagents should be directed to and will be fulfilled by the Lead Contact, Qi Cui (qi.cui@pennmedicine.upenn.edu).

#### Materials Availability

This study did not generate new unique reagents. However, there are some restrictions to the availability of the *Il1a*^−*/*−^ *Tnf*^−*/*−^ *C1qa*^−*/*−^ mouse line due to MTAs. Please contact Qi Cui (qi.cui@pennmedicine.upenn.edu) for more information.

#### Data and Code Availability

This study did not generate or utilize any dataset or code that requires distribution.

### EXPERIMENTAL MODEL AND SUBJECT DETAILS

#### Mice

All mice were adult (> 3 months old), age-, strain- and sex-matched (both male and female mice were used in each analysis, in all groups). C57BL6/J (WT) mice were obtained from Jackson labs (Stock Number 000664). *Il1a*−*/*−*; Tnf*−*/*−, *C1qa*−*/*−, and *Il1a*−*/*−*; Tnf*−*/*−*; C1qa* −*/*− (TKO) animals were generously donated by Ben Barres (Stanford University). All animals were fed *ad libitum* and maintained on a 12 h/12 h light/dark cycle in a University of Pennsylvania vivarium. All procedures were approved by the Institutional Animal Care and Use Committee of the University of Pennsylvania and complied with the ARVO Statement for the Use of Animals in Ophthalmic and Vision Research. NLY01 was obtained through a material transfer agreement with Neuraly (Baltimore, MD). A cohort of mice was treated with either twice-weekly subcutaneous injections of NLY01 (5 mg kg^−1^ per injection) or with an equivalent amount of normal saline solution (L21819; Fisher Scientific).

### METHOD DETAILS

#### Anterior Chamber Injection and Intraocular Pressure (IOP) Measurement

The microbead occlusion model was used to induce elevated intraocular pressure as described previously (Cui et al., 2020). Briefly, mice were anesthetized with intraperitoneal injections of ketamine (80 mg/kg, Par Pharmaceutical), xylazine (10 mg/kg, Lloyd), and acepromazine (2 mg/kg, Boehringer Ingelheim Vetmedica). Pupils were dilated with topical 1% tropicamide and 2.5% phenylephrine (Akorn). Proparacaine anesthetic eye drop at a concentration of 0.5% (Sandoz) were applied immediately prior to injection. Injection micropipettes were pulled from glass capillaries to a final diameter of ~100 μm and connected to a microsyringe pump. Using a micromanipulator for positioning, one eye of the mouse was injected with 1.5 μL of sterile 4.5 μm-diameter magnetic microbeads (1.6 × 10^6^ beads/μL of balanced salt solution; Thermo Fisher Scientific) at a location < 1 mm central to the limbus, while the other eye was injected with an equivalent volume of balanced salt solution (Alcon Laboratories). A hand-held magnet was used to target the beads into the drainage angle. After injection, 0.5% moxifloxacin antibiotic drops (Sandoz) was applied to the eye. IOP was measured between 8 and 11 a.m. using the Icare TONOLAB tonometer (Icare TONOVET). An average of three measurements/eye was used.

#### Retinal cell sorting

ACSA2^+^, CD11b^+^ CD11c^−^, and CD11b^+^ CD11c^+^ cells were isolated from adult murine retinas using the Miltenyi Adult Brain Dissociation Kit (Miltenyi Biotec, 130-107-677) and MACS magnetic cell separation system. Briefly, mice were anesthetized, euthanized, and whole neurosensory retinas were harvested. Retinal tissue was dissociated in manufacturer provided enzyme mixtures using the gentleMACS dissociator pre-set program: 37C_ABDK_02. Retinal cell suspensions were passed through a 70 μm filter (130-098-462, Miltenyi Biotec) and resuspended in debris removal solution. Following debris removal, retinal cells were resuspended and incubated in red blood cell removal solution for 10 mins at 4°C. The following isolation steps were performed: (1) positive selection for ACSA2 (astrocyte and Muller cell enriched, 130-097-678, Miltenyi Biotec), (2) the remaining negative selection pool was then subjected to positive selection for CD11b (microglia and macrophage enriched, 130-093-636 Miltenyi Biotec), and (3) the CD11b^+^ cells were then selected against CD11c (130-125-835, Miltenyi Biotec). This resulted in three cell populations: (1) ACSA2^+^, (2) ACSA2^−^ CD11b^+^ CD11c^−^, and (3) ACSA2^−^ CD11b^+^ CD11c^+^. Positive and negative selection steps used MACS magnetic separation LS columns (130-042-401, Miltenyi Biotech) according to the manufacturer’s protocol. Cells were subsequently used for RNA isolation using the RNeasy Mini Kit (QIAGEN), according to the manufacturer’s protocol.

#### Quantitative PCR (qPCR)

RNA isolation was performed according to the manufacturer’s protocol (RNeasy kit; QIAGEN). cDNA was synthesized with reverse transcription agents (TaqMan Reverse Transcription Reagents, Applied Biosystems) according to the manufacturer’s protocol. Real-time qPCR (TaqMan; ABI) was performed on a sequence detection system (Prism Model 7500; ABI) using the ΔΔCT method, which provided normalized expression values (normalized against *Gapdh*). All reactions were performed in technical triplicates (three qPCR replicates per qPCR probe).

#### Enzyme-linked immunosorbent assays

ELISA kits were used to measure protein levels of IL-1α (BMS627, Thermofisher Scientific), TNF-α (BMS607-3, Thermofisher Scientific), C1q (LS-F55223-1, LifeSpan BioSciences), C3 (ab157711, Abcam), and phospho- NFκB/total NFκB (Thermofisher Scientific, 50-246-259) according to the manufacturer’s protocol. Briefly, mouse retinas were collected, in select cases subjected to cell sorting, and subsequently homogenized in phosphate-buffered saline containing the protease inhibitor phenylmethylsulfonylfluoride (100 lM; EMD, Gibbstown, NJ, USA). Assays were performed according to the manufacturers’ protocol. Protein levels were determined by comparing the absorbance produced by the samples with that of a calibration curve. All measurements were performed in technical triplicate.

#### Preparation of retinal flatmounts, immunofluorescence, and cell counting

RGC quantification and immunolabeling of flat-mounted retinas were performed as previously described (Cui et al., 2020). Briefly, eyes were enucleated and fixed in 4% paraformaldehyde. Retinas were isolated, mounted on glass slides and serially washed with 0.5% Triton X-100 in phosphate-buffered saline (PBS). Flat-mounted retinas were incubated overnight at 4°C with antibodies against RNA-binding protein with multiple splicing (RBPMS; EMD Millipore) diluted 1:500 in blocking buffer (2% bovine serum albumin, 2% Triton X-100 in PBS) and brain-specific homeobox/POU domain protein-3a (Brn3a; Synaptic Systems) diluted 1:1000 in blocking buffer. The following day, retinas were washed and incubated with Alexa Fluor 488 donkey anti-rabbit IgG (Invitrogen; 1:1000 in blocking buffer) and Cy3 goat anti-guinea pig IgG (Abcam; 1:500 in blocking buffer) secondary antibodies for 3 hours at room temperature. After serial washes, flat-mounts were coverslipped with Vectashield mounting medium containing DAPI (Vector Laboratories). For each flat-mount, 12 standardized photomicrographs were taken at 1/6, 3/6, and 5/6 distance from the center of the retina at 40× magnification by a masked operator. A masked counter quantified the number of Rbpms- and Brn3a-positive cells in each 40× field (0.069 mm^2^) using Nikon Elements analysis software version 4.1 (Nikon Instruments). The average number of cells in the 12 standardized photomicrographs from the microbead-injected eye was normalized to the average number of cells in the 12 standardized photomicrographs from the BSS-injected eye to calculate percent survival in the microbead injected eye with eIOP.

### QUANTIFICATION AND STATISTICAL ANALYSIS

All statistical analyses were done using GraphPad Prism 8.0 software. Data were analyzed either by one-way ANOVA followed by Tukey’s multiple comparisons test for comparing between three or more samples, or Mann-Whitney U test for comparing between two samples with 95% confidence without assuming a Gaussian distribution. Samples sizes and p-values can be found in figure legends. Power calculations were performed using G* Power Software V 3.1.9.7 (Faul et al., 2007). Group sizes were calculated to provide at least 80% power with the following parameters: probability of type I error (0.05), effect size (0.25).

## Supplementary Material

1

2

## Figures and Tables

**Table:** KEY RESOURCES TABLE

REAGENT or RESOURCE	SOURCE	IDENTIFIER
Antibodies		
Guinea pig polyclonal anti-RBPMS	EMD Millipore	Cat# ABN1376; RRID:AB_2687403
Rabbit polyclonal anti-Brn3a	Synaptic systems	Cat# 411 003; RRID: AB_2737037
Donkey anti-rabbit IgG, Alexa Fluor 488	Invitrogen	Cat# A-21206; RRID: AB_2535792
Goat anti-guinea pig IgG, Cy3	Abcam	Cat# ab102370; RRID: AB_10711466
Chemicals, Peptides, and Recombinant Proteins		
NLY01	Neuraly	N/A
Normal saline solution	Fisher Scientific	Cat# B21819
Tropicamide ophthalmic solution, 1%	Akorn	NDC# 17478-102-12
Phenylephrine hydrochloride ophthalmic solution, 2.5%	Akorn	NDC# 17478-201-15
Ketamine hydrochloride injection	Par Pharmaceutical	NDC# 42023-113-10
Xylazine sterile solution	Lloyd	NADA# 139-236
Proparacaine hydrochloride ophthalmic solution, 0.5%	Sandoz	NDC# 61314-016-01
Acepromazine	Boehringer Ingelheim Vetmedica	NDC# 0010-6703-01
Balanced salt solution sterile irrigating solution	Alcon Laboratories	NDC# 0065-0795-15
Moxifloxacin ophthalmic solution, 0.5%	Sandoz	DIN# 02411520
Phenylmethylsulfonylfluoride	EMD Millipore	CAS# 329-98-6
Triton X-100	Millipore Sigma	Cat# T8787
Bovine serum albumin	Amresco	Cat# 0332-25G
Paraformaldehyde	Electron Microscopy Sciences	Cat# 15710
Vectashield mounting medium with DAPI	Vector laboratories	Cat# H-1200
Critical Commercial Assays		
IL-1α Rat ELISA Kit	ThermoFisher Scientific	Cat# BMS627
TNF-α Mouse ELISA Kit	ThermoFisher Scientific	Cat# BMS607-3
Mouse Complement C1q ELISA Kit	LifeSpan Biosciences	Cat# LS-F55223-1
Mouse Complement C3 ELISA Kit	Abcam	Cat# ab157711
NFkB p65 (Total/Phospho) Human InstantOne ELISA Kit	ThermoFisher Scientific	Cat# 85-86083-11
RNeasy Mini Kit	QIAGEN	Cat# 74106
Adult Brain Dissociation Kit	Miltenyi Biotec	Cat# 130-107-677
Experimental Models: Organisms/Strains		
Mouse: C57BL/6J	The Jackson Laboratory	JAX:000664
Mouse: *Il1a*^−/−^;*Tnf*^−/−^	Ben Barres, Stanford University	N/A
Mouse: *C1qa*^−/−^	Ben Barres, Stanford University	N/A
Mouse: *Il1a*^−/−^;*Tnf*^−/−^;*C1qa*^−/−^	Guttenplan et al., 2020	N/A
Software and Algorithms		
G* Power Software version 3.1.9.7	Faul et al., 2007	https://stats.idre.ucla.edu/other/gpower/
Applied Biosystems 7500 Real-Time PCR	TaqMan, Applied Biosystems	Cat# 4351105
Nikon Elements Analysis Software 4.1	Nikon Instruments	N/A
Other		
Dynabeads M-450 Epoxy	ThermoFisher Scientific	Cat# 14011
Icare TONOLAB tonometer	Icare TONOVET	Cat# TV02
MACS SmartStrainer filter (70 mm)	Miltenyi Biotec	Cat# 130-098-462
CD11b (Microglia) MicroBeads, human and mouse	Miltenyi Biotec	Cat# 130-093-636
CD11c MicroBeads UltraPure, mouse	Miltenyi Biotec	Cat# 130-125-835
Anti-ASCA2 MicroBead Kit, mouse	Miltenyi Biotec	Cat# 130-097-678

## Highlights

- Astrogliosis contributes to retinal ganglion cell death in a mouse glaucoma model
- CD11b^+^ CD11c^+^ cells contribute to astrogliosis after ocular hypertension
- Reduction of inflammatory cytokines protects against astrogliosis and cell death
- NLY01, a GLP-1R agonist, prevents astrogliosis and retinal ganglion cell death

## References

[R1] Aroda VR (2018). A review of GLP-1 receptor agonists: evolution and advancement, through the lens of randomized controlled trials. Diabetes Obes. Metab 20 (Suppl 1), 22–33. 29364586 10.1111/dom.13162

[R2] Athauda D, and Foltynie T (2016). The glucagon-like peptide 1 (GLP) receptor as a therapeutic target in Parkinson’s disease: mechanisms of action. Drug Discov. Today 21, 802–818. 26851597 10.1016/j.drudis.2016.01.013

[R3] Bennett ML, Bennett FC, Liddelow SA, Ajami B, Zamanian JL, Fernh-off NB, Mulinyawe SB, Bohlen CJ, Adil A, Tucker A, (2016). New tools for studying microglia in the mouse and human CNS. Proc. Natl. Acad. Sci. USA 113, E1738–E1746. 26884166 10.1073/pnas.1525528113 PMC4812770

[R4] Bosco A, Anderson SR, Breen KT, Romero CO, Steele MR, Chiodo VA, Boye SL, Hauswirth WW, Tomlinson S, and Vetter ML (2018). Complement C3-targeted gene therapy restricts onset and progression of neurodegeneration in chronic mouse glaucoma. Mol. Ther 26, 2379–2396. 30217731 10.1016/j.ymthe.2018.08.017 PMC6171099

[R5] Bozkurt B, Mesci L, Irkec M, Ozdag BB, Sanal O, Arslan U, Ersoy F, and Tezcan I (2012). Association of tumour necrosis factor-alpha −308 G/A polymorphism with primary open-angle glaucoma. Clin. Exp. Ophthalmol 40, e156–e162. 21575121 10.1111/j.1442-9071.2011.02595.x

[R6] Buckingham BP, Inman DM, Lambert W, Oglesby E, Calkins DJ, Steele MR, Vetter ML, Marsh-Armstrong N, and Horner PJ (2008). Progressive ganglion cell degeneration precedes neuronal loss in a mouse model of glaucoma. J. Neurosci 28, 2735–2744. 18337403 10.1523/JNEUROSCI.4443-07.2008 PMC6670674

[R7] Calkins DJ, Lambert WS, Formichella CR, McLaughlin WM, and Sappington RM (2018). The Microbead Occlusion Model of Ocular Hypertension in Mice. Methods Mol. Biol 1695, 23–39. 29190015 10.1007/978-1-4939-7407-8_3

[R8] Clarke LE, and Barres BA (2013). Emerging roles of astrocytes in neural circuit development. Nat. Rev. Neurosci 14, 311–321. 23595014 10.1038/nrn3484 PMC4431630

[R9] Cui QN, Bargoud AR, Ross AG, Song Y, and Dunaief JL (2020). Oral administration of the iron chelator deferiprone protects against loss of retinal ganglion cells in a mouse model of glaucoma. Exp. Eye Res 193, 107961. 32045598 10.1016/j.exer.2020.107961 PMC7584350

[R10] Daruich A, Matet A, Moulin A, Kowalczuk L, Nicolas M, Sellam A, Rothschild P-R, Omri S, Gélizé E, Jonet L, (2018). Mechanisms of macular edema: beyond the surface. Prog. Retin. Eye Res 63, 20–68. 29126927 10.1016/j.preteyeres.2017.10.006

[R11] Drucker DJ (2018). Mechanisms of action and therapeutic application of glucagon-like peptide-1. Cell Metab. 27, 740–756. 29617641 10.1016/j.cmet.2018.03.001

[R12] Fan BJ, Liu K, Wang DY, Tham CCY, Tam POS, Lam DSC, and Pang CP (2010). Association of polymorphisms of tumor necrosis factor and tumor protein p53 with primary open-angle glaucoma. Invest. Ophthalmol. Vis. Sci 51, 4110–4116. 20357201 10.1167/iovs.09-4974

[R13] Faul F, Erdfelder E, Lang A-G, and Buchner A (2007). G*Power 3: a flexible statistical power analysis program for the social, behavioral, and biomedical sciences. Behav. Res. Methods 39, 175–191. 17695343 10.3758/bf03193146

[R14] Guttenplan KA, Stafford BK, El-Danaf RN, Adler DI, Münch AE, Weigel MK, Huberman AD, and Liddelow SA (2020). Neurotoxic reactive astrocytes drive neuronal death after retinal injury. Cell Rep. 31, 107776. 32579912 10.1016/j.celrep.2020.107776 PMC8091906

[R15] Howell GR, Macalinao DG, Sousa GL, Walden M, Soto I, Kneeland SC, Barbay JM, King BL, Marchant JK, Hibbs M, (2011). Molecular clustering identifies complement and endothelin induction as early events in a mouse model of glaucoma. J. Clin. Invest 121, 1429–1444. 21383504 10.1172/JCI44646 PMC3069778

[R16] Howell GR, MacNicoll KH, Braine CE, Soto I, Macalinao DG, Sousa GL, and John SWM (2014). Combinatorial targeting of early pathways profoundly inhibits neurodegeneration in a mouse model of glaucoma. Neurobiol. Dis 71, 44–52. 25132557 10.1016/j.nbd.2014.07.016 PMC4319373

[R17] Ito YA, Belforte N, Vargas JLC, and Polo AD (2016). A magnetic microbead occlusion model to induce ocular hypertension-dependent glaucoma in mice. J. Vis. Exp (109), e53731. 27077732 10.3791/53731 PMC4841308

[R18] Kantzer CG, Boutin C, Herzig ID, Wittwer C, Reiß S, Tiveron MC, Drewes J, Rockel TD, Ohlig S, Ninkovic J, (2017). Anti-ACSA-2 defines a novel monoclonal antibody for prospective isolation of living neonatal and adult astrocytes. Glia 65, 990–1004. 28317180 10.1002/glia.23140

[R19] Kaur C, Foulds WS, and Ling EA (2008). Blood-retinal barrier in hypoxic ischaemic conditions: basic concepts, clinical features and management. Prog. Retin. Eye Res 27, 622–647. 18940262 10.1016/j.preteyeres.2008.09.003

[R20] Khan A, Petropoulos IN, Ponirakis G, and Malik RA (2017). Visual complications in diabetes mellitus: beyond retinopathy. Diabet. Med 34, 478–484. 27917530 10.1111/dme.13296

[R21] Kokona D, Ebneter A, Escher P, and Zinkernagel MS (2018). Colony-stimulating factor 1 receptor inhibition prevents disruption of the blood-retina barrier during chronic inflammation 15, 340. 10.1186/s12974-018-1373-4 PMC6292111 30541565

[R22] Lambert WS, Carlson BJ, Formichella CR, Sappington RM, Ahlem C, and Calkins DJ (2017). Oral delivery of a synthetic sterol reduces axonopathy and inflammation in a rodent model of glaucoma. Front. Neurosci 11, 45. 28223915 10.3389/fnins.2017.00045 PMC5293777

[R23] Lambert WS, Pasini S, Collyer JW, Formichella CR, Ghose P, Carlson BJ, and Calkins DJ (2020). Of mice and monkeys: neuroprotective efficacy of the p38 inhibitor BIRB 796 depends on model duration in experimental glaucoma. Sci. Rep 10, 8535. 32444682 10.1038/s41598-020-65374-6 PMC7244559

[R24] Lee NY, Kim MH, and Park CK (2017). Visual field progression is associated with systemic concentration of macrophage chemoattractant protein-1 in normal-tension glaucoma. Curr. Eye Res 42, 1002–1006. 28306361 10.1080/02713683.2016.1276193

[R25] Liddelow SA, and Barres BA (2017). Reactive astrocytes: production, function, and therapeutic potential. Immunity 46, 957–967. 28636962 10.1016/j.immuni.2017.06.006

[R26] Liddelow SA, Guttenplan KA, Clarke LE, Bennett FC, Bohlen CJ, Schirmer L, Bennett ML, Münch AE, Chung W-S, Peterson TC, (2017). Neurotoxic reactive astrocytes are induced by activated microglia. Nature 541, 481–487. 28099414 10.1038/nature21029 PMC5404890

[R27] Margeta MA, Lad EM, and Proia AD (2018). CD163^+^ macrophages infiltrate axon bundles of postmortem optic nerves with glaucoma. Graefes Arch. Clin. Exp. Ophthalmol 256, 2449–2456. 30073622 10.1007/s00417-018-4081-y PMC6221945

[R28] Mookherjee S, Banerjee D, Chakraborty S, Banerjee A, Mukhopadhyay I, Sen A, and Ray K (2010). Association of IL1A and IL1B loci with primary open angle glaucoma. BMC Med. Genet 11, 99. 20565898 10.1186/1471-2350-11-99 PMC2909939

[R29] Nadal-Nicolás FM, Jiménez-López M, Sobrado-Calvo P, Nieto-López L, Cánovas-Martínez I, Salinas-Navarro M, Vidal-Sanz M, and Agudo M (2009). Brn3a as a marker of retinal ganglion cells: qualitative and quantitative time course studies in naive and optic nerve-injured retinas. Invest. Ophthalmol. Vis. Sci 50, 3860–3868. 19264888 10.1167/iovs.08-3267

[R30] Quigley HA (2019). 21st century glaucoma care. Eye (Lond.) 33, 254–260. 30305707 10.1038/s41433-018-0227-8 PMC6367343

[R31] Reinehr S, Reinhard J, Gandej M, Kuehn S, Noristani R, Faissner A, Dick HB, and Joachim SC (2016). Simultaneous complement response via lectin pathway in retina and optic nerve in an experimental autoimmune glaucoma model. Front. Cell. Neurosci 10, 140. 27313510 10.3389/fncel.2016.00140 PMC4887475

[R32] Risner ML, Pasini S, Cooper ML, Lambert WS, and Calkins DJ (2018). Axogenic mechanism enhances retinal ganglion cell excitability during early progression in glaucoma. Proc. Natl. Acad. Sci. USA 115, E2393–E2402. 29463759 10.1073/pnas.1714888115 PMC5877940

[R33] Risner ML, McGrady NR, Pasini S, Lambert WS, and Calkins DJ (2020). Elevated ocular pressure reduces voltage-gated sodium channel NaV1.2 protein expression in retinal ganglion cell axons. Exp. Eye Res 190, 107873. 31734278 10.1016/j.exer.2019.107873 PMC6957720

[R34] Sappington RM, Carlson BJ, Crish SD, and Calkins DJ (2010). The microbead occlusion model: a paradigm for induced ocular hypertension in rats and mice. Invest. Ophthalmol. Vis. Sci 51, 207–216. 19850836 10.1167/iovs.09-3947 PMC2869054

[R35] Stasi K, Nagel D, Yang X, Wang R-F, Ren L, Podos SM, Mittag T, and Danias J (2006). Complement component 1Q (C1Q) upregulation in retina of murine, primate, and human glaucomatous eyes. Invest. Ophthalmol. Vis. Sci 47, 1024–1029. 16505037 10.1167/iovs.05-0830

[R36] Stevens B, Allen NJ, Vazquez LE, Howell GR, Christopherson KS, Nouri N, Micheva KD, Mehalow AK, Huberman AD, Stafford B, (2007). The classical complement cascade mediates CNS synapse elimination. Cell 131, 1164–1178. 18083105 10.1016/j.cell.2007.10.036

[R37] Tham Y-C, Li X, Wong TY, Quigley HA, Aung T, and Cheng C-Y (2014). Global prevalence of glaucoma and projections of glaucoma burden through 2040: a systematic review and meta-analysis. Ophthalmology 121, 2081–2090. 24974815 10.1016/j.ophtha.2014.05.013

[R38] Tribble JR, Harder JM, Williams PA, and John SWM (2019). Suppression of homeostatic gene expression and increased expression of metabolism-related genes are early features of glaucoma in optic nerve head microglia. bioRxiv. 10.1101/856427.

[R39] Tribble JR, Harder JM, Williams PA, and John SWM (2020a). Ocular hypertension suppresses homeostatic gene expression in optic nerve head microglia of DBA/2 J mice. Mol. Brain 13, 81. 32450896 10.1186/s13041-020-00603-7 PMC7249412

[R40] Tribble JR, Kokkali E, Otmani A, Plastino F, Lardner E, Vohra R, Kolko M, André H, Morgan JE, and Williams PA (2020b). When is a control not a control? Reactive microglia occur throughout the control contralateral visual pathway in experimental glaucoma. bioRxiv. 10.1101/853275. PMC7804521 33510961

[R41] Vecino E, Rodriguez FD, Ruzafa N, Pereiro X, and Sharma SC (2016). Glia-neuron interactions in the mammalian retina. Prog. Retin. Eye Res 51, 1–40. 26113209 10.1016/j.preteyeres.2015.06.003

[R42] Wang C-Y, Shen Y-C, Lo F-Y, Su C-H, Lee S-H, Lin K-H, Tsai H-Y, Kuo N-W, and Fan S-S (2006). Polymorphism in the IL-1alpha (−889) locus associated with elevated risk of primary open angle glaucoma. Mol. Vis 12, 1380–1385. 17149369

[R43] Williams PA, Tribble JR, Pepper KW, Cross SD, Morgan BP, Morgan JE, John SWM, and Howell GR (2016). Inhibition of the classical pathway of the complement cascade prevents early dendritic and synaptic degeneration in glaucoma. Mol. Neurodegener 11, 26. 27048300 10.1186/s13024-016-0091-6 PMC4822272

[R44] Williams PA, Marsh-Armstrong N, and Howell GR; Lasker/IRRF Initiative on Astrocytes and Glaucomatous Neurodegeneration Participants (2017). Neuroinflammation in glaucoma: a new opportunity. Exp. Eye Res 157, 20–27. 28242160 10.1016/j.exer.2017.02.014 PMC5497582

[R45] Williams PA, Braine CE, Kizhatil K, Foxworth NE, Tolman NG, Harder JM, Scott RA, Sousa GL, Panitch A, Howell GR, and John SWM (2019). Inhibition of monocyte-like cell extravasation protects from neurodegeneration in DBA/2J glaucoma. Mol. Neurodegener 14, 6. 30670050 10.1186/s13024-018-0303-3 PMC6341618

[R46] Yun SP, Kam T-I, Panicker N, Kim S, Oh Y, Park J-S, Kwon S-H, Park YJ, Karuppagounder SS, Park H, (2018). Block of A1 astrocyte conversion by microglia is neuroprotective in models of Parkinson’s disease. Nat. Med 24, 931–938. 29892066 10.1038/s41591-018-0051-5 PMC6039259

[R47] Zamanian JL, Xu L, Foo LC, Nouri N, Zhou L, Giffard RG, and Barres BA (2012). Genomic analysis of reactive astrogliosis. J. Neurosci 32, 6391–6410. 22553043 10.1523/JNEUROSCI.6221-11.2012 PMC3480225

