# Supplementary material for: GLP-1 Receptor Agonist NLY01 Reduces Retinal Inflammation and Neuron Death Secondary to Ocular Hypertension
Source: Cell Rep. Author manuscript; Available in PMC 2020 Nov 12. (PMC7660987; doi:10.1016/j.celrep.2020.108271)
Supplement: 1 [file NIHMS1643866-supplement-1.pdf]

Cell Reports, Volume *33*

Supplemental Information

GLP-1 Receptor Agonist NLY01 Reduces Retinal Inflammation and Neuron Death Secondary to Ocular Hypertension

Jacob K. Sterling, Modupe O. Adetunji, Samyuktha Guttha, Albert R. Bargoud, Katherine E. Uyhazi, Ahmara G. Ross, Joshua L. Dunaief, and Qi N. Cui

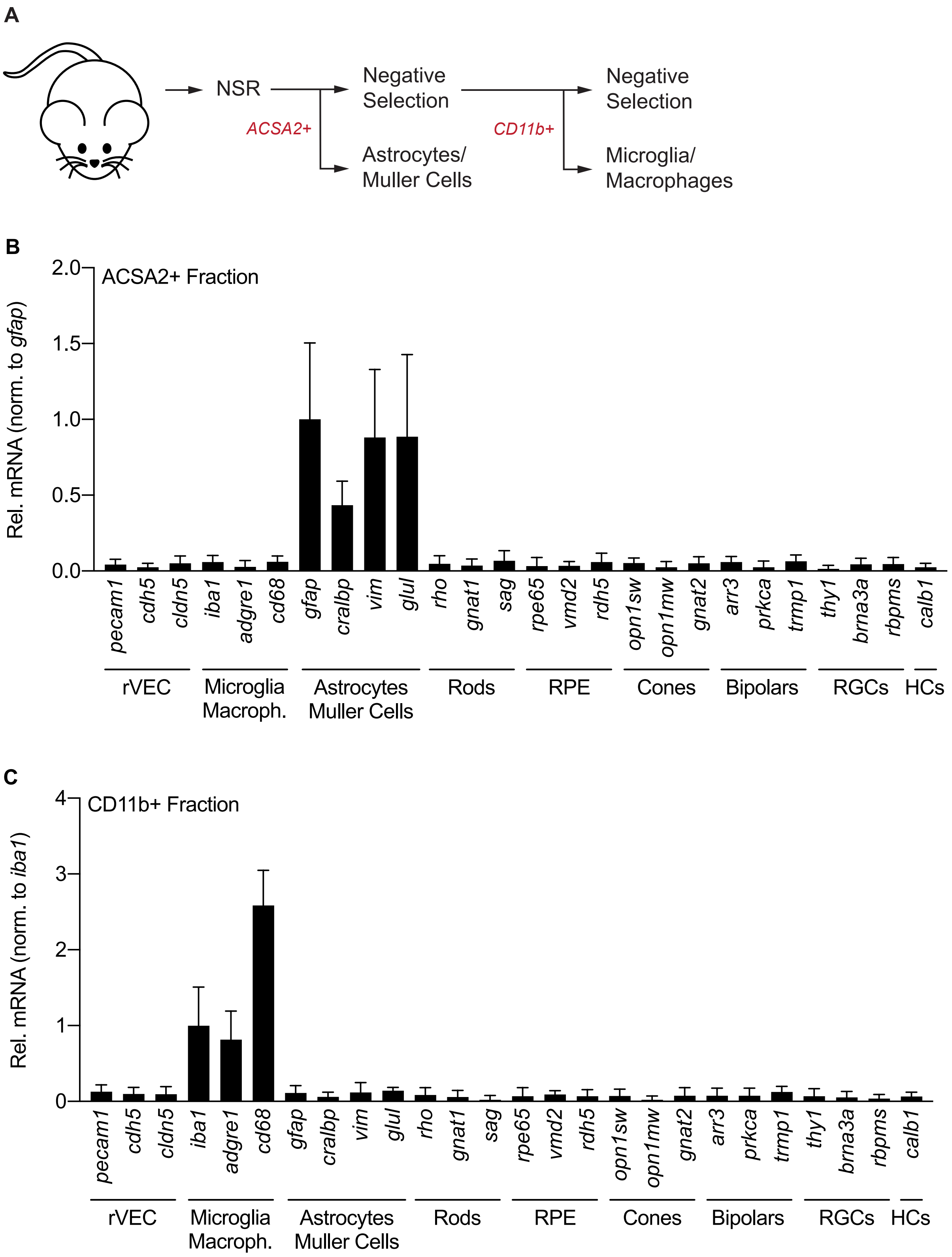

Supplemental Figure 1. Cell sorting paradigm. Related to Figures 1, 3, 4, and 5.
C57BL6/J (WT) mice were euthanized and neurosensory retina (“NSR”) was isolated and dissociated for cell sorting.
(A) Cell isolation protocol using magnetic cell sorting.
(B) qPCR measurements of cell-type specific markers in ACSA2+ cells.
(C) qPCR measurements of cell-type specific markers in CD11b+ cells.
n = 8 eyes. All data presented as mean ± SEM.

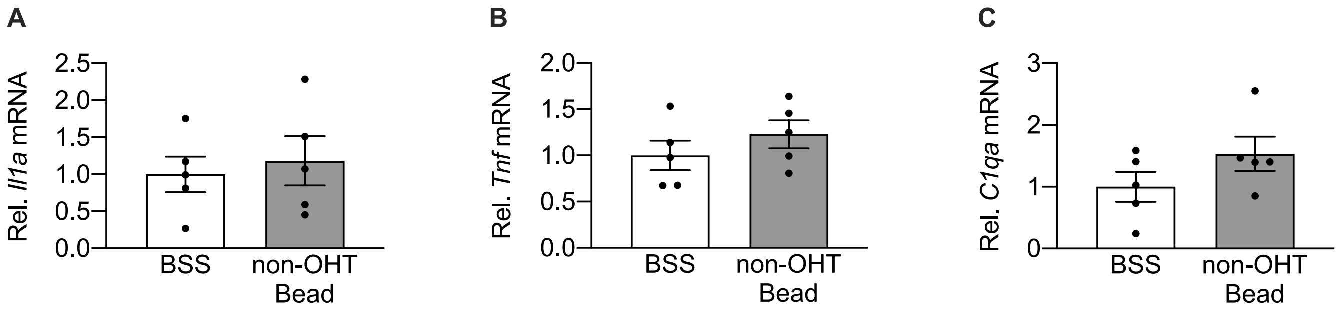

Supplemental Figure 2. Microbead-injection alone does not induce CD11b+ production of Il1a, Tnf, or C1qa. Related to Figure 3.
C57BL6/J (WT) mice were injected either with microbeads (left eye), to increase intraocular pressure (IOP), or with BSS (right eye). IOP was monitored weekly. Bead eyes that did not have an IOP increase of 6 mmHg or greater within 2 weeks of injections were termed “non-OHT Bead” and excluded from eIOP studies.
(A-C) CD11b+ cells were isolated from neurosensory retina 42 days after injection. qPCR was performed to measure Il1a (A), Tnf (B), and C1qa (C) mRNA levels.
n=5 eyes per condition. All data presented as mean ± SEM.

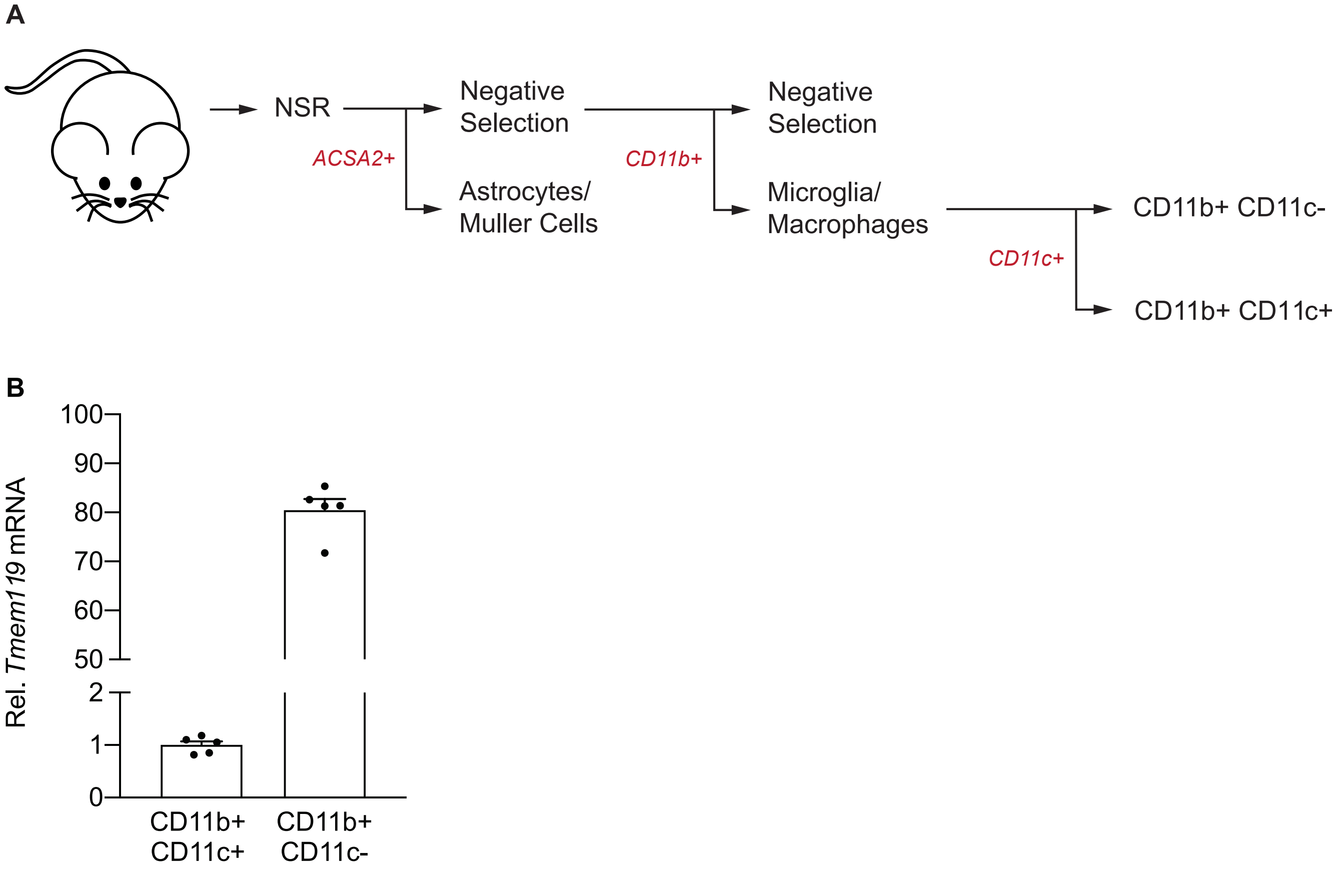

Supplemental Figure 3. CD11b+ CD11c- vs CD11b+ CD11c+ cell sorting paradigm. Related to Figure 3. C57BL6/J (WT) mice were euthanized and neurosensory retina ("NSR") was isolated and dissociated for cell sorting.

(A) Cell isolation protocol using magnetic cell sorting.

(B) qPCR measurement of *Tmem119* mRNA in CD11b+ CD11c+ cells vs. CD11b+ CD11c- cells.

n = 5 eyes. All data presented as mean ± SEM.

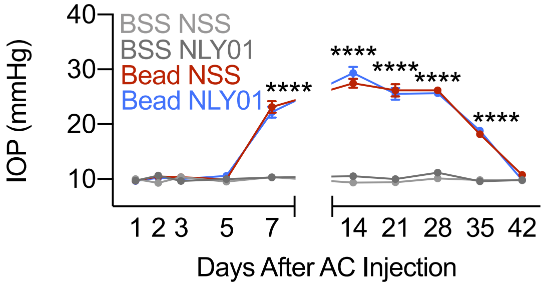

Supplemental Figure 4. NLY01 does not affect intraocular pressure. Related to Figures 4, 5 and 6. C57BL6/J (WT) mice were injected either with microbeads ("Bead", left eye), to increase intraocular pressure (IOP), or with BSS (right eye). Following intraocular injections, mice were randomized to twice weekly sub-cutaneous NLY01 (5 mg kg-1 per injection) or normal saline solution (NSS). IOP were measured across the duration of the study. Statistical difference for Bead NSS vs BSS NSS and Bead NLY01 vs BSS NYL01 shown using stars. No statistical difference was detected between BSS NSS and BSS NLY01 or between Bead NSS and Bead NLY01. All data presented as mean ± SEM. Mann-Whitney U test, ****$p<0.0001$. (n = 25 eyes per condition per treatment)

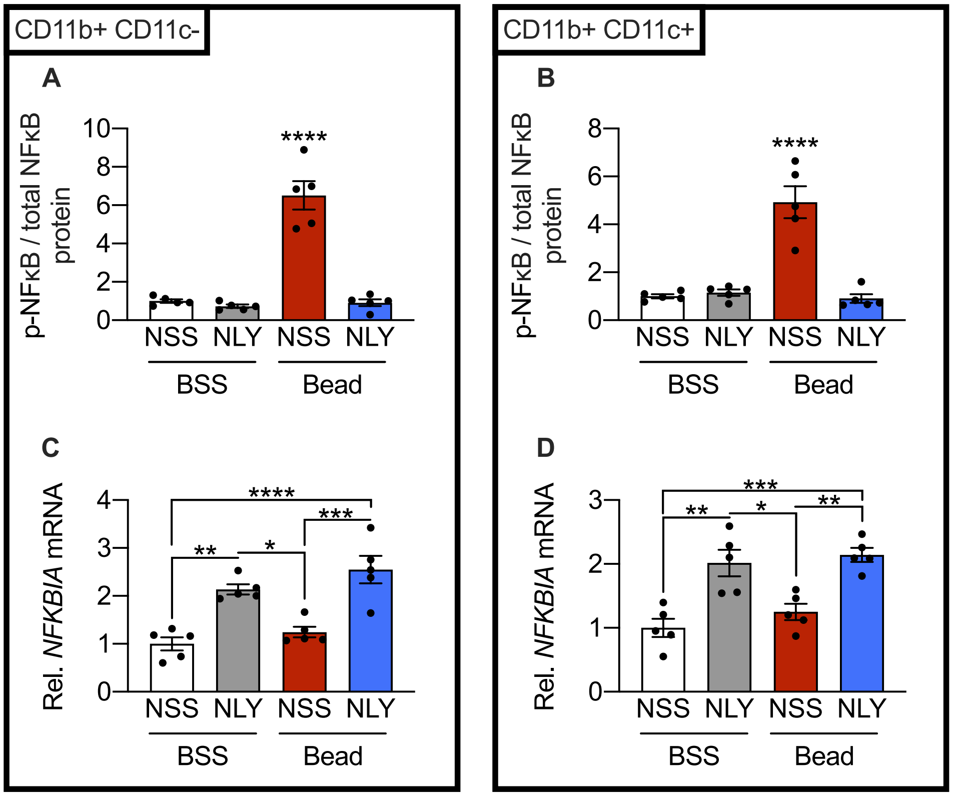

Supplemental Figure 5. NLY01 modulates NFκB signaling in CD11b+ CD11c- and CD11b+ CD11c+ cells. Related to Figures 4 and 5.

C57BL6/J (WT) mice were injected either with microbeads (“Bead”, left eye), to increase intraocular pressure (IOP), or with BSS (right eye). Following intraocular injections, mice were randomized to twice weekly subcutaneous NLY01 (5 mg kg-1 per injection) or normal saline solution (NSS). 42 days post-injection mice were euthanized, neurosensory retina was harvested and sorted as described in Fig. S3A to isolate CD11b+ CD11c- and CD11b+ CD11c+ cells.

(A) ELISA measurements of phospho-NFκB normalized to total NFκB in CD11b+ CD11c- cells.

(B) ELISA measurements of phospho-NFκB normalized to total NFκB in CD11b+ CD11c+ cells.

(C) qPCR measurement of NFKBIA mRNA levels in CD11b+ CD11c- cells.

(D) qPCR measurement of NFKBIA mRNA levels in CD11b+ CD11c+ cells.

All data presented as mean ± SEM. Ordinary one-way ANOVA, $^*p<0.05$, $^{**}p<0.01$, $^{***}p<0.001$, $^{****}p<0.0001$.
